# A Retrospective Digital Analysis of Contour Changing after Tooth Extraction with or without Using Less Traumatic Surgical Procedures

**DOI:** 10.3390/jcm11040922

**Published:** 2022-02-10

**Authors:** Giovanni Battista Menchini-Fabris, Paolo Toti, Roberto Crespi, Giovanni Crespi, Saverio Cosola, Ugo Covani

**Affiliations:** 1 Department of Stomatology, Tuscan Stomatologic Institute, Foundation for Dental Clinic, Research and Continuing Education, 55041 Camaiore, Italy; capello.totipaolo@gmail.com (P.T.); robcresp@libero.it (R.C.); gio.crespi@hotmail.it (G.C.); s.cosola@hotmail.it (S.C.); covani@covani.it (U.C.); 2 Study Center for Multidisciplinary Regenerative Research, Guglielmo Marconi University, 00100 Rome, Italy; 3 San Rossore Dental Unit, Viale delle Cascine 152, San Rossore, 56122 Pisa, Italy; 4 Department of Dentistry, Unicamillus—Saint Camillus International University of Health and Medical Sciences, 00100 Rome, Italy

**Keywords:** alveolar remodeling, tooth extraction, intraoral digital scanning, imaging superimposition, less traumatic surgery, socket healing

## Abstract

Background: The present retrospective analysis aimed to compare two different single tooth extraction surgical approaches in both premolar and molar areas: less traumatic magneto-electrical versus conventional tooth extraction in minimizing the edentulous ridge volume loss. Methods: In the present retrospective control trial, 48 patients who underwent one-tooth extraction, were allocated either to control (28 sites treated with conventional tooth extraction procedures) or test group (20 subjects treated with less traumatic tooth extraction procedures by tooth sectioning and magnetoelectric roots subluxation). Intraoperatively (during tooth extraction surgery just after the subsequent filling of the alveolar socket with the sterile fast re-absorbable gelatin sponge), and then four months later, contours of the sockets were acquired through a laser intra-oral scanner. The digitally superimposed models were converted to dicom (Digital Imaging and Communications in Medicine) format first, then volumetric and area evaluations were performed with a DentaScan tool package. Non-parametric tests were applied with a level of significance set at *p* < 0.01. Results: significant reductions of anatomical features were observed four months later in all the groups (*p*-values < 0.001) with volume losses leading to a final alveolar ridge volume of 0.87 ± 0.34 cm^3^ for atraumatic extractions and 0.66 ± 0.19 cm^3^ for conventional extractions. No significant differences were registered for outcomes related to the basal surface variables. When just molar tooth were considered, the outcomes relating to volume loss between baseline and four months (ΔV) and its percentage (ΔV%) showed a better behavior in the less traumatic procedure (ΔV = −0.30 ± 0.10 cm^3^ and ΔV% = −22.3 ± 8.4%) compared to the conventional extractions (ΔV = −0.59 ± 0.10 cm^3^ and ΔV% = −44.3 ± 5.8%) with *p*-values < 0.0001. Conclusions: at four months, the less traumatic tooth extraction procedures by tooth sectioning and magnetoelectric root subluxation seemed to be able to better preserve the volume of the alveolar crest (reduction close to 22% with less traumatic extraction in molar sites) when compared to subjects treated with the conventional tooth extraction techniques.

## 1. Introduction

It was well known that to place a dental implant reaching an acceptable aesthetics of prosthetic restoration, it is fundamental to manage the alveolar bone remodeling after tooth extraction by counteracting the reduction of width and height of the alveolar ridge [1,2,3].

The remodeling of hard and soft tissues could be affected by many different factors, such as the anatomical features of the extraction sites, all the other treatments following the extraction surgery, and obviously, any surgical procedure or tooth extraction technique as well [4,5,6,7].

To minimize any negative impact of the tooth removal procedure to the alveolar socket healing, several instruments had been introduced and used during the so-called Less Traumatic Extraction Techniques” (LTETs) such as forceps, periotomes, and luxators, along with piezosurgical, magnetoelectrical, and root extraction system devices [8].

Conventional extraction surgery consisted in using the elevators and forceps, which could easily damage the coronal aspect of the buccal and palatal/lingual cortical bone of the alveolar crest; this occurred if shattered root fragments had to be removed with the reflection of a mucoperiosteal flap, with the removal of bone to retrieve roots, and by utilizing tooth movement in a horizontal direction or by rotating it till to root(s) luxation [9,10].

In this respect, elevators could pull out the tooth from a socket by using adjacent bone margins acting as fulcra [11]. This high extractive force used could cause severe soft and hard tissue trauma [12].

When more aggressive surgeries had to be used, i.e., for multi-rooted teeth with ankilotic or divergent roots, different minimally invasive procedures that applied a mechanical strength rather than using the force of the surgeon had been described [13].

In this view, any damage caused to the facial bone wall of the alveolar socket at the time of extraction could influence the loss in width and height of the alveolar ridge during the healing period. They were, precisely, the piezosurgical devices and vibrating syndesmotomes that gently acted to sever the cervical fibers of the periodontal ligament surrounding the tooth between the root and socket. So, all this ensured that the coronal tissues of the extraction socket did not undergo any traumatic ripping [14,15].

The alveolar shrinkage after tooth extraction was so well known that clinicians devised several methods for maintaining or augmenting the ridge volume waiting for delayed implant placement [16,17]. Different grafting materials and techniques were recommended to preserve the alveolar ridge during the healing phase [18,19,20]. However, a clinician who was very careful when handling the tissues rounding a tooth to be extracted played an important part in the alveolar ridge preservation.

The concept behind root extraction systems was that a single root could be pulled out in its axial direction with precision given by the several proposed corkscrew devices without any direct trauma to the socket walls [21]. This strategy was of particular relevance in single-rooted teeth (anterior maxilla and mandible). On the contrary, since no extractions of teeth in posterior sites could adversely affect aesthetic outcomes, it was reported that the buccal contour of the alveolar ridge underwent 50% volume loss within one year after surgery [1].

A less traumatic tooth extraction could be performed by the clinician even without the aid of any device or new technology. As said, electromagnetic dental mallet helped reduce tissues damage in implant prosthetic rehabilitation as suggested by Crespi and co-workers [22]. A midcourse between very less traumatic devices and surgeon manual intervention could be the use of mechanical periotomes that advanced apically with minimal hand pressure in a quick and precise way and without any effort of the clinicians in extracting teeth [23].

Three-dimensional digital systems employed in the rehabilitation workflow, such as digital models as an alternative to plaster casts, represented an important technological advancement allowing identification of better surgical procedures and translating the adoption of more effective therapies [24,25]. Stereolithographic (.stl) model allowed the clinicians to calculate the changes guaranteeing high levels of accuracy when different .stl point clouds had to be superimposed [16,26]. This could be carried out semi-automatically with the help of a clinician (via triangulation of the occlusal planes) [27].

The primary aim of the present retrospective analysis was to test the effectiveness of two types of posterior single tooth extraction (less traumatic magneto-electrical versus conventional tooth extraction) in maintaining contour stability of the socket area; sockets were observed using an intraoral laser scanner that provided three-dimensional digital models of the patients’ dental arches acquired intraoperatively (just after tooth extraction) and then four months after the first surgery. A secondary aim was to test if a loss in the contour of the edentulous area depended on the extracted tooth site (bicuspids versus molars).

## 2. Materials and Methods

### 2.1. Study Design/Sample

Case sheets of patients treated for tooth extraction from 2016 to 2019 were gathered to access patients’ personal information. Collected schedules were reviewed to extract useful information and relevant data.

Patients inclusion criteria:

- 18 years old or older;
- signed and informed consent form for data processing;
- single intercalate tooth extraction in the back area (bicuspid and molar teeth);
- presence of an uncorrupted dataset of two three-dimensional scans (file.stl) in the collected records, representing intraoperative views on just treated sites (acquired during tooth extraction surgery just after the filling of the alveolar socket with a sterile fast re-absorbable gelatin sponge) and on healed postsurgical areas (around 4 months later).

Patients exclusion criteria:

- history of systemic diseases contraindicating oral surgical intervention;
- any report for bisphosphonate therapy;
- history of bone resection or radiation therapy (as part of an oncological treatment);
- lost or corrupted .stl file of the virtual models.

Patients were intra- and postoperatively scanned with a 25 µm precision 3-dimensional optical scanner (TRIOS 3, 3Shape A/S, Holmens Kanal, Copenhagen, Denmark).

A matrix elaborator (MatLab 7.11, The MathWorks, Natick, MA, USA) read information from .stl files and processed data of two full-arch digital models. For each patient, digital stereolithographic files were voxelized; the process of voxelization consisted of converting the .stl vertices into the same number of voxels to create 16-bit three-dimensional clouds; their primary characteristic was that they could be easily read on the dentascan (list in Appendix A). The two voxelized clouds were superimposed each other by using a best-fit algorithm as described and listed by Menchini-Fabris and co-workers to occupy the same space at the same time; the position of each digital model was triangulated from its occlusal surface given by the remaining teeth to be exact [27]; then the matrices were fused each other by another subroutine (list in Appendix B).

Results were saved as dicom images by applying the following setting: Field Of View = 10.24 cm, isometric voxel = 100 µm.

Dicom images with fused full-arch digital models underwent volume and surface measurements in a dedicated dentascan software (SimPlant 12.02, Materialise Dental Italia, Roma, Italy) as per Crespi and colleague [28].

**Figure 1:**
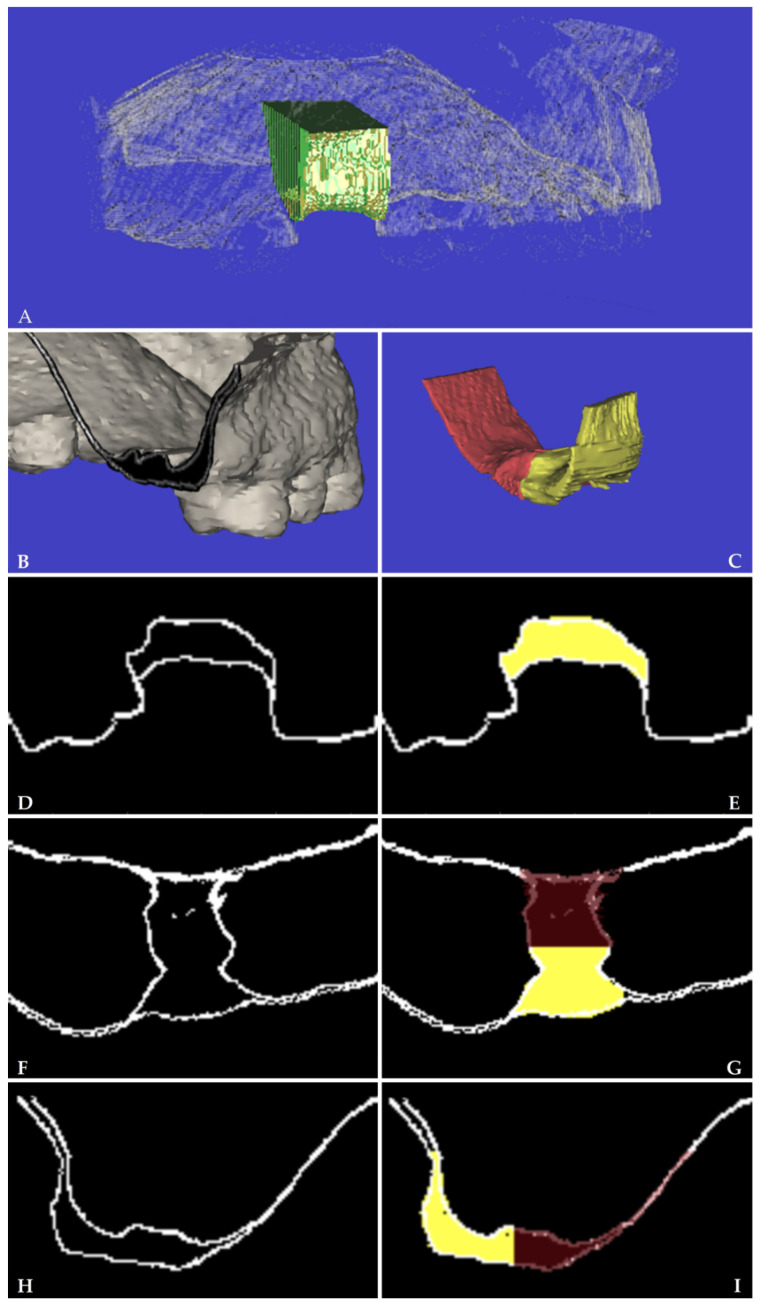
Buccal and palatal volume measurements (**A**) Three-dimensional voxelized .stl cloud with measured alveolar ridge volume at T0 (in green) and basal surface (in dark green) within the Volume Of Interest, (**B**) isometric rendering of two fused voxelized .stl clouds (intraoperative versus 4-month survey) with (**C**) view of the change of alveolar ridge volume, buccal (in yellow) and palatal (in red). (**D**,**E**) sagittal, (**F**,**G**) axial, and (**H**,**I**) cross-sectional views of the clouds obtained by an intraoral optical scanner.

The boundaries of the standardized Volume Of Interest (VOI) were defined as the following. VOI domain was a parallelepiped with six faces: mesial and distal border walls were perpendicular to both a cross-sectional line passing in the middle of the alveolar ridge and the occlusal plane, and they were tangential to the remaining teeth surfaces towards the edentulous area (distal crown surface of the anterior tooth and mesial crown surface of the posterior tooth, respectively); buccal/palatal border walls were perpendicular to both the mesial and the distal walls, as well as to the occlusal plane; basal/coronal walls were perpendicular to all the others being, respectively, the base and the cover of the VOI box. Coronal boundary stretched from the most coronal point of preoperative papillae to the level of 10 mm toward the apical direction, which corresponded to the basal plane (or surface). A graphical representation of the VOI was shown in Figure 1.

Then, a single-blind examiner and collector (TP) performed all volume and area measurements using the “prepare for planning” toolbox of the dentascan.

### 2.2. Surgery Procedures

One hour before surgery, patients were treated with “one-shot” antibiotic administration as a pre-medication (2 g amoxicillin or 0.6 g. of clindamycin for subjects allergic to penicillins and cephalosporins). After a mouth rinse with 0.2% chlorhexidine for 1 min, patients were treated under local anesthesia using lidocaine with epinephrine (1:50,000).

Less traumatic tooth extraction

The tooth was extracted with the maximum preservation of the hard and soft tissue with the least traumatic procedure as possible. The way was to pull out each tooth just like a single-rooted one. When multi-rooted, the tooth required a surgical crown sectioning with one root per crown segment, on the other hand in the cases of fused or convergent roots, sectioning was not required. Neither flaps were raised nor releasing incisions performed. When necessary periotomes were used to sever the cervical gingival attachment fibers. Extraction was performed using an electromagnetic device (Magnetic Mallet, www.osseotouch.com (accessed on 6 January 2022), Turbigo, Milano, Italy) that applied on the tip of the thin metallic blade a calibrated shock wave of 130 daN. The longitudinal movements imparted by the device promoted the penetration of the blade parallel to the long axis of the tooth (or each root) advancing apically in 2mm increments at both mesial and distal aspects with minimal hand pressure.

After applying the magnetoelectric device, each tooth/root could be easily removed without applying any latero-lateral force with luxators pushing in forward/rearward and upwards direction and with extraction forceps for residual roots exerting rotational force in a coronal direction.

Conventional tooth extraction

After clinical assessment of tooth to be extracted, periosteal elevators were used for reflecting the gingiva to expose the cemento-enamel junction and the extraction was carried out using conventional forceps and luxating elevators by dislodging the tooth without tooth sectioning, as per a simple extraction (that is, an intact tooth removal) without any mechanical device. No force other than manually was used to extract the tooth. Neither flaps were raised nor releasing incisions was performed.

Subsequently, for both groups a sterile re-absorbable gelatin sponge (Cutanplast^®^ Dental, Dispotech S.r.l., Gordona (SO), Italy) was placed to fill the socket and secured with sutures. Sutures were used to stabilize collagen and blood clots.

Immediately after the surgery and domiciliary for oneweek, patients were asked to apply an oral amino-acids based gel with hyaluronic acid (Aminogam gel^®^ of Polifarma Benessere S.r.l., Rome, Italy) after the oral hygiene procedures to reduce swelling and pain.

### 2.3. Outcomes

Descriptive variables were registered: age, gender, smoking habits, and tooth location.

Primary predictor variable

- test group “*ltr*”, less traumatic tooth extraction; control group “*con*”, conventional tooth extraction.
- Secondary predictor variable
- Tooth site: premolar versus molar; aspect: buccal versus palatal

Primary outcome variables

The measurer calculated anatomical variables based on volumetric and superficial features of the extraction site and expressed in cm^3^/cm^2^ to two decimal places. All anatomical measurements were positive. 

**Figure 2:**
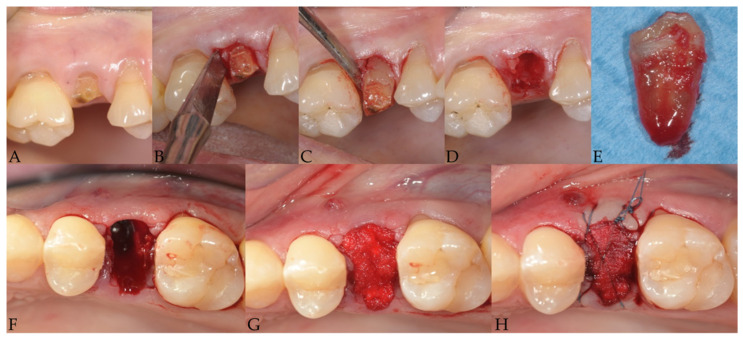
Clinical view of a less extraction technique. (**A**) preoperative; (**B**,**C**) electromagnetic tips for mesial and distal luxation; (**D**) extraction; (**E**) extracted tooth; (**F**) post extraction socket; (**G**) gelatin sponge; (**H**) sutures.

V_T0_ and V_T1_: volume of the alveolar ridge within the standardized VOI, respectively, at the intraoperative time point (T0) and 4 months after tooth extraction (T1) (Figure 2).

BS_T0_ and BS_T1_: basal surface of the alveolar ridge or the area of the most apical axial) of the VOI box, respectively, at T0 and T1 (Figure 1).

Secondary outcome variables

All outcomes were obtained by a series of algebraic manipulations of the primary ones. The secondary outcomes were usually negative and represented a loss in volume or a reduction in surface. 

Volume change of the alveolar ridge from T0 to T1, or ΔV (evaluated by subtracting the baseline value V_T0_ from that of the intraoperative survey V_T1_) and its analogous in terms of percentage within the VOI, were respectively given by Equations (1) and (2):

(1) $$\Delta V=V_{T1}-V_{T0}$$

(2) $$\Delta V\%=100\cdot \left(V_{T1}-V_{T0}\right)/V_{T0}$$

Change at basal surface with its loss in terms of percentage were given by Equations (3) and (4)

(3) $$\Delta \mathrm{BS}={\mathrm{BS}}_{T1}-{\mathrm{BS}}_{T0}$$

(4) $$\Delta \mathrm{BS}\%=100\cdot \left({\mathrm{BS}}_{T1}-{\mathrm{BS}}_{T0}\right)/V_{T0}$$

### 2.4. Statistical Analysis

A statistician performed all analyses using a statistical tool from a Matrix Laboratory (Statistics Toolbox, MatLab 7.11; The MathWorks, Natick, MA, USA).

There was one extraction site per patient so that the two groups were independent; normal distribution for each outcome variable was checked, but not confirmed, by the Shapiro–Wilk test [29]. Moreover, the assumption of homoscedasticity for equality of variances was not met by Brown-Forsythe’s test for all groups and subgroups investigated.

Wilcoxon tests were employed for pair-wise comparisons for matched and unmatched samples; Spearman’s correlation assessed the strength of the bivariate association between the outcomes and the other variables.

The effects of the sample and the results of the power analysis were, respectively, determined with a power of 0.99, the reported sample size, and both measures of central tendency and dispersion.

The level of statistical significance was set at 0.01 for all analyses.

## 3. Results

In the present analysis, 48 patients were considered eligible. All the demographic data and variables’ descriptions and dispersions about the extracted teeth ranked between the groups had been reported in Table 1. Healing following tooth extraction in 45 sites appeared uneventful; three sites showed swelling, redness, and flow of exudate resolved within one week of adjunctive antibiotic administration, as shown in Table 1.

### 3.1. Primary Predictors: Procedures

**Figure 3:**
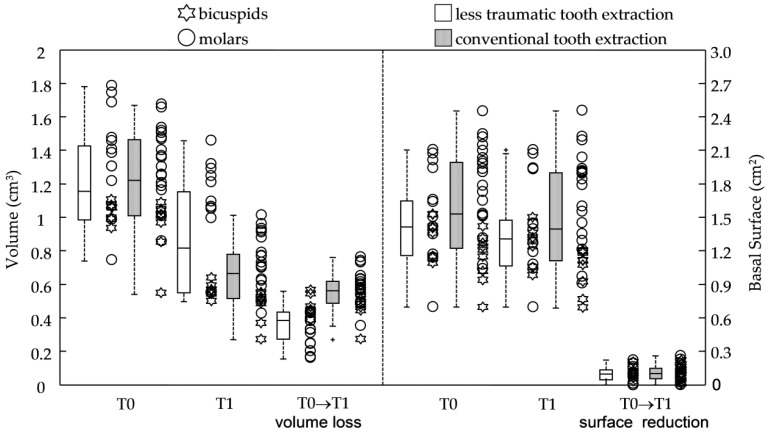
Scatter and box plots for the anatomical variables and outcomes at baseline (T0) and 4month postoperative time (T1) in *ltr* and *con* group; volumes V, basal surfaces BS, and their change at 3 months. In the box-and-whiskers plot, the box line represents the lower, median, and upper quartile values, the whisker lines include the rest of the data. Outliers (+) were data with values beyond the ends of the whiskers. Scatter data were ranked by tooth type.

The two groups were of similar sizes in terms of pristine surface area and baseline volume, while significant reductions of anatomical features were observed four months later in all the groups (Table 1 and Figure 3 with *p*-values ≤ 0.0002). In fact, in both groups, the volume losses (−0.36 ± 0.12 cm^3^ and −0.56 ± 0.11 cm^3^, respectively, for *ltr* and *con* group) and reduction of the basal surfaces (−0.10 ± 0.07 cm^2^ for both of them) were registered at four-month follow-up, leading to a final alveolar ridge volume of 0.87 ± 0.34 cm^3^ for less traumatic extractions and 0.66 ± 0.19 cm^3^ for conventional extractions. No significant differences were registered for outcomes related to the basal surface variables. 

Correlation analyses between each secondary outcome and all the anatomical variables were shown in Table 2. No significant correlations were reported for *ltr* group. In the conventional extraction group, the outcome related to volume resorption (ΔV) had a negative correlation with both the pristine volume (*r_s_*−0.7588 with *p*-value < 0.0001), and basal surface at baseline (*r_s_*−0.7122 with *p*-value < 0.0001).

### 3.2. Secondary Predictors: Tooth Aspect and Site

When extraction types were investigated for buccal or palatal/lingual aspects, the observed behaviors were similar to those recorded in the previous section and shown in Table 3; that is, significant differences had been recorded between *ltr* and *con* groups regarding the percentages of volume loss (with ranges from 28.6 to 34.4% and from 41.5 to 52.8%, respectively, for less traumatic and conventional procedure) with *p*-values ≤ 0.0046. Again, all the anatomical variables (V and BS) were significantly different between the two aspects (*p*-value ≤ 0.0004), but just the outcome ΔV% showed a higher rate in the conventional group when buccal (−52.8 ± 7.3%) and palatal aspect (−41.5 ± 8.4%) had been compared (*p*-value < 0.0001).

When premolar and molar sites had been evaluated, the type of extraction showed a small impact on the volume loss; in fact, ΔVs and ΔV%s were, respectively, −0.46 ± 0.06 cm^3^ and −44.9 ± 4.2% for the less traumatic group and −0.47 ± 0.08 cm^3^ and −50.0 ± 3.4% for the conventional group without any significant differences.

However, when just molar tooth were considered, analysis of outcomes relating to the volume showed a better behavior in the less traumatic procedure (ΔV = −0.30 ± 0.10 cm^3^ and ΔV% = −22.3 ± 8.4%) when compared to the conventional extractions (ΔV = −0.59 ± 0.10 cm^3^ and ΔV% = −44.3 ± 5.8%) with *p*-values < 0.0001.

## 4. Discussion

The purpose of this retrospective control study was to test the effectiveness of posterior single tooth extraction with or without a less traumatic extraction procedure in preserving existing alveolar ridge contours of the fresh socket using an intraoral laser scanner. Intraoperative digital cast model was compared to that of the healed site obtained four months after tooth extraction, before rehabilitation with implant-supported fixed single crown prosthesis. The stereolithographic files were voxelized and digitally superimposed by a matrix laboratory. Detailed analyses of contour modifications were performed on two fused voxelized .stl clouds.

It was often difficult to precisely define the meaning of an “atraumatic extraction” when considering the wide variety of described extraction techniques. As said, nevertheless, most of described atraumatic procedures with or without the use of several and special tools could certainly cause less damage to the tissues surrounding teeth, but, to a certain degree, still traumatized the bone to some extent [7,30].

So, all the other conventional or experimental extracting procedures, according to this view, could be defined as traumatic or less-traumatic ones. There was no question that any force application in horizontal directions could affect, in single-rooted teeth, alveolar bone remodeling more than rotational movements, so the application of forces in the buccal/palatal directions was much worse than those in the mesial/distal directions [31].

The final point needed to describe a less traumatic extraction technique was the use of any device whose primary function was breaking of periodontal fibers and removing conical roots without overexpansion of the alveolar socket.

The energy translated by the magnetoelectrical device into pulse pressure, which moved the subluxating periotome blade applied a vertical compressive and penetrating force along the root length detached the root from the surrounding alveolar tissues, and left intact the bony plate. Once each root was subluxated, it could be pulled out by using forceps for residual extraction of dental roots in a simple rotational movement [12].

Surgical sectioning was required when it appeared necessary to convert a posterior tooth into a multiple “single-rooted” one. On the contrary, in the event of fused or convergent roots, a multiple rooted tooth could be removed without sectioning [23].

The present study suggested that alveolar ridges of less traumatic extraction group reported at the four-month survey significantly (*p*-value = 0.0001) lower volume loss (31.3%) versus those treated with conventional traumatic extraction procedures with forceps and luxators (46.2%). This was true for both the aspects (buccal and lingual/palatal), even if just for volumetric outcomes. In the present study, non-significant dimensional changes were observed in the basal surface, with a small reduction registered in the volume of interest (decrease ranging from 5.6 and 8.6%). This was in line with evidence-based information reported in the literature on the factors affecting ridge width and height modification after tooth removal such as a flap or flapless technique, smoking habit, drugs administration during healing, number and shape of roots, and the status of the buccal bony plate (thin or fenestrated) [14,32,33].

Results regarding the behavior of alveolar bone remodeling in posterior areas were scanty. However, some studies attested that naturally healing sites that underwent tooth extraction showed a loss in height ranging from 1.4 to 3.6 mm and a reduction in width ranging from 2.3 to 4.5 mm irrespective of tooth site [34,35,36,37]. Some studies suggested that ridge preservation using low resorbing xenograft could considerably limit the amount of horizontal ridge resorption when compared with tooth extraction alone: a difference ranged between the two groups from −3.33 to −2 mm [19,38]. However, no information was provided regarding the type of extraction (more or less traumatic). When changes in the volume of the post-extraction sites underwent no socket ridge preservation were investigated, Sbordone and co-workers found that ridge preservation compensated for the postextraction alveolar ridge resorption with a loss of about 22% in the external contour [16]. Whereas, when clinicians left the extraction socket undisturbed, this might result in an alveolar contour shrinkage close to 40% after three to four months [16]. However, a less traumatic tooth extraction could counteract a volume loss of 10% leading to a volume loss of −31.3% as reported in the present findings. Moreover, when subgroups related to the tooth site were analyzed separately, molars of the less traumatic group suffered a significantly smaller loss in terms of volume outcomes (22.3%) than that of the traumatic extractions (44.3%). This did not happen in the bicuspid areas. Tooth type (bicuspids versus molars) seemed to influence the magnitude of the three-dimensional (3D) shape changes when less traumatic extraction had been performed in the posterior areas.

In this view, tooth extraction without damaging the hard and soft tissues of the post extractive socket was just the first step. Some authors suggested that the preservation of the socket volume was dependent first and foremost on maintaining pristine volume during extraction and then on clothing the socket to prevent contact between the healing tissues and the intraoral environment. This could be achieved not by filling the post-extraction socket with slow resorbing materials, but rather by using a tooth-like emergence profile when an immediate implant had been placed or by using an immediate pontic (very similar to the emergence profile of the natural tooth). With advances in three-dimensional printing, the use of materials as biocompatible as possible had offered new clinical opportunities. These new techniques could be used to produce scaffold for tissues’ reconstruction with a highly precise and accurate design [39] or to fabricate any structure of mechanical interest in dentistry, which appeared to be individualized for each patient (for example surgical guides and orthodontic power-arms) [40,41].

The limitations of the present study might be an error generated during the acquisition of the arch digital impression. The presence of blood and spit during the production of the digital cast could be a primary source of the inaccuracy of the present optical scanning technique. No extrapolation could be made as to whether the volume resorption was caused by loss of soft tissue or underlying bone. Finally, the small number of patients/casts in each group might be another bias that could affect the measurement of true effectiveness, in terms of the percentages of loss of the external contours.

The use of a magnetoelectrical device probably minimized mechanical impacts on the alveolar tissues resulting in a reduction in volume two times that of sites with more traumatic tooth extraction, as the combined result of teeth segmentation and roots subluxation. In comparison with other conventional techniques for less traumatic tooth extraction, the magnetoelectric device played the same role as the periotomes, but with an additional feature of mesial/distal subluxation. Moreover, an advantage when using the magnetoelectric devices was that the instrument produced less heat and requires less cooling than the conventional rotary cutting, sonosurgery, piezosurgery, and piezoelectric devices [42,43].

However, it might be said that the present study included teeth with no buccal or palatal/lingual bone defects involving the alveolar crest. Thus, it is important to note that the applicability and results of the present procedure are not directly extensible to such severely damaged alveolar sockets.

## 5. Conclusions

The four-month analysis test group showed a reduced loss of the external contour when compared to the conventional tooth extraction technique. However, the less traumatic procedures seemed to be able to better preserve the volume of the alveolar crest (reduction close to 22% with less traumatic extraction) even if just for molars.

Tooth position (bicuspids versus molars) seemed to affect volume loss but not shrinkage of the basal surface with the molar site generally favored in volume preservation.

## Figures and Tables

**Table 1:** Demographic data and homogeneity analysis between the two groups, less traumatic tooth extraction (*ltr*) and conventional tooth extraction (*con*), with descriptive variables (gender male/female, Y/N, and bicuspids/molars ratio, Bd/Mr and swelling events Y/N). The assumption of homoscedasticity was not met by Brown-Forsythe’s test for equality of variances F = 4.7130, df1 = 3, df2 = 92, *p* = 0.0042, and F = 4.6245, df1 = 3, df2 = 92, *p* = 0.0047 for overall volume variable and its buccal aspect. Bd, bicuspid; Mr, molar. Anatomical and outcome variables at baseline (T0) and at 4 months (T1, when the site was healed): volume of the alveolar ridge or V, basal surface or BS, and outcome variables (alveolar ridge volume and basal change percentages, respectively, ΔV% and BS%. Shapiro–Wilk test significance (*p_SW_*); Wilcoxon rank-sum test significance between unpaired data (*p_Wu_*); Wilcoxon signed-rank test significance between paired data (*p_Wp_*); Results of Fischer test (*p_F_*). Statistically-significant values are in bold. Report of calculated sample size (with power = 0.99) and calculated power.

Group	Less Traumatic Tooth Extraction (*ltr*)			Conventional Tooth Extraction (*con*)			*p*_F_ *ltr* vs. *con*			
sample size	20			28			-			
genders F/M	12/8			15/13			0.7710			
Ratio Bd/Mr	8/12			9/19			0.7603			
Smoke Y/N	2/18			2/26			1.0000			
age (range)	53.4 ± 8.2 (41.0–70.0)			46.0 ± 10.9 (25.1–63.7)			-			
swelling Y/N	1/19			2/26			1.0000			
**primary predictor: experimental groups**										
	**N = 20**		***p*_Wp_ times**	**N = 28**		***p*_Wp_ times**	***p*_Wu_** ***ltr* vs. *con***		***ltr* vs. *con***	
**Time X**	**T0**	**T1**	**T0 vs. T1**	**T0**	**T1**	**T0 vs. T1**	**T0**	**T1**	**sample size**	**power**
V (cm^3^)	1.22 ± 0.29	0.87 ± 0.34	**<0.0001**	1.22 ± 0.27	0.66 ± 0.19	**<0.0001**	0.9084	0.0346		
*p*_SW_ (SW)	0.2156	**0.0031**		0.4917	0.5950					
BS(cm^2^)	1.47 ± 0.37	1.37 ± 0.38	**<0.0001**	1.59 ± 0.47	1.49 ± 0.49	**<0.0001**	0.4207	0.6157		
*p*_SW_ (SW)	0.3829	0.0983		0.5656	0.2511					
ΔV (cm^3^)		−0.36 ± 0.12			−0.56 ± 0.11			**<0.0001**	17	1.00
*p*_SW_ (SW)		0.3050			0.3042					
ΔV%		−31.3 ± 13.3			−46.2 ± 5.8			**0.0001**	21	0.99
*p*_SW_ (SW)		0.0472			0.5962					
ΔBS(cm^2^)		−0.10 ± 0.07			−0.10 ± 0.07			0.7458	N.D	0.00
*p*_SW_ (SW)		0.3581			0.3531					
ΔBS%		−6.8 ± 4.5			−7.2 ± 5.4			0.9583	7807	0.01
*p*_SW_ (SW)		0.0909			0.1936					

**Table 2:** Spearman’s correlation coefficients *r_s_* with significances between outcome variables and overall anatomical variables for alveolar ridge modification in two groups, less traumatic tooth extraction (*ltr*) and conventional tooth extraction (*con*).

	Procedure	Less Traumatic Tooth Extraction (*ltr*)			Conventional Tooth Extraction (*con*)		
Outcome Variables	vs.	$V_{T0}$	$BS_{T0}$	$iH_{T0}$	$V_{T0}$	$BS_{T0}$	$iH_{T0}$
ΔV	correlation coefficient (*r*_s_)	0.3179	0.0188	0.4664	−0.7588	−0.7122	0.4412
	significance (two-tailed)	0.1720	0.9373	0.0313	**<0.0001**	**<0.0001**	0.0188
ΔBS	correlation coefficient (*r*_s_)	0.1695	0.1061	0.1203	0.2436	0.2573	−0.3038
	significance (two-tailed)	0.4750	0.6562	0.6133	0.2115	0.1863	0.1160

**Table 3:** Anatomical variables and outcomes for the group less traumatic tooth extraction (*ltr*) and conventional tooth extraction (con), at baseline (T0) and at 4 months (T1, when the site was healed) for the secondary predictor tooth aspect and location: buccal versus palatal and bicuspid versus molar. Volume of alveolar ridge or V, basal surface or BS, and outcome variables (percentages of Volume and Basal Surface change). Shapiro–Wilk test significance (*p_SW_*); Wilcoxon rank-sum test significance between unpaired data (*p_Wu_*); Wilcoxon signed-rank test significance between paired data (*p_Wp_*). Statistically-significant values are in bold. Report of calculated sample size (with power = 0.99) and calculated power.

Group	Less Traumatic Tooth Extraction (*ltr*)			Conventional Tooth Extraction (*con*)						
**secondary predictor: buccal**										
	**N = 20**		***p*_Wp_** **Times**	**N = 28**		***p*_Wp_** **Times**	***p*_Wu_** ***ltr* vs. *con***		***ltr* vs. *con***	
**Time X**	**T0**	**T1**	**T0 vs. T1**	**T0**	**T1**	**T0 vs. T1**	**T0**	**T1**	**sample Size**	**power**
V (cm^3^)	0.52 ± 0.15	0.35 ± 0.15	**<0.0001**	0.51 ± 0.14	0.24 ± 0.09	**<0.0001**	0.9084	0.0196		
*p*_SW_ (SW)	0.1979	0.0434		0.2889	0.2471					
BS(cm^2^)	0.58 ± 0.14	0.53 ± 0.13	**0.0003**	0.65 ± 0.21	0.60 ± 0.21	**<0.0001**	0.2415	0.2499		
*p*_SW_ (SW)	0.9000	0.7803		0.1869	0.1184					
ΔV (cm^3^)		−0.17 ± 0.05			−0.26 ± 0.07			**<0.0001**	24	0.97
*p*_SW_ (SW)		0.6222			0.1246					
ΔV%		−34.4 ± 13.1			−52.8 ± 7.3			**<0.0001**	150	0.22
*p*_SW_ (SW)		0.8268			0.8452					
ΔBS(cm^2^)		−0.05 ± 0.04			−0.05 ± 0.04			0.6444	N.D	0.00
*p*_SW_ (SW)		0.1914			0.0143					
ΔBS%		−8.6 ± 6.7			−7.8 ± 6.4			0.6671	3268	0.01
*p*_SW_ (SW)		0.2789			0.0456					
**secondary predictor:** **palatal**										
	**N = 20**		***p*_Wp_** **times**	**N = 28**		***p*_Wp_** **times**	***p*_Wu_** ***ltr* vs. *con***		***ltr* vs. *con***	
**Time X**	**T0**	**T1**	**T0 vs. T1**	**T0**	**T1**	**T0 vs. T1**	**T0**	**T1**	**sample size**	**power**
V (cm^3^)	0.71 ± 0.17	0.52 ± 0.19	**<0.0001**	0.71 ± 0.16	0.42 ± 0.13	**<0.0001**	0.9666	0.1549		
*p*_SW_ (SW)	0.0826	0.0598		0.1458	0.9979					
BS(cm^2^)	0.89 ± 0.30	0.84 ± 0.32	**0.0002**	0.94 ± 0.29	0.89 ± 0.30	**<0.0001**	0.4771	0.6605		
*p*_SW_ (SW)	0.1706	0.0687		0.4612	0.2866					
ΔV (cm^3^)		−0.20 ± 0.10			−0.29 ± 0.08			**0.0014**	48	0.72
*p*_SW_ (SW)		0.5916			0.3528					
ΔV%		−28.6 ± 15.1			−41.5 ± 8.4			**0.0046**	149	0.22
*p*_SW_ (SW)		0.0289			0.7460					
ΔBS(cm^2^)		−0.04 ± 0.04			−0.06 ± 0.05			0.3270	261	0.11
*p*_SW_ (SW)		**0.0089**			0.0129					
ΔBS%		−5.6 ± 5.2			−6.7 ± 6.1			0.5203	1338	0.02
*p*_SW_ (SW)		**0.0021**			**0.0085**					
***p*_Wp_ between buccal and palatal**										
**Time X**	**T0**	**T1**		**T0**	**T1**					
V (cm^3^)	**0.0003**	**0.0001**		**<0.0001**	**<0.0001**					
BS(cm^2^)	**0.0002**	**0.0001**		**<0.0001**	**<0.0001**					
ΔV (cm^3^)		0.3134			0.0855					
ΔV%		0.0206			**<0.0001**					
ΔBS(cm^2^)		0.3812			0.2584					
ΔBS%		0.1024			0.6567					
**secondary predictor: bicuspids**										
	**N = 8**		***p*_Wp_** **times**	**N = 9**		***p*_Wp_** **times**	***p*_Wu_** ***ltr* vs. *con***		***ltr* vs. *con***	
**Time X**	**T0**	**T1**	**T0 vs. T1**	**T0**	**T1**	**T0 vs. T1**	**T0**	**T1**	**sample size**	**power**
V (cm^3^)	1.02 ± 0.06	0.56 ± 0.04	**0.0078**	0.95 ± 0.17	0.47 ± 0.09	**0.0039**	0.4650	**0.0058**		
*p*_SW_ (SW)	0.6037	0.3417		**0.0030**	**0.0090**					
BS(cm^2^)	1.33 ± 0.18	1.23 ± 0.18	**0.0078**	1.14 ± 0.22	1.03 ± 0.19	**0.0039**	0.1455	0.0879		
*p*_SW_ (SW)	0.1687	0.5479		0.5263	0.0577					
ΔV (cm^3^)		−0.46 ± 0.06			−0.47 ± 0.08			0.3203	2597	0.01
*p*_SW_ (SW)		0.2554			0.0134					
ΔV%		−44.9 ± 4.2			−50.0 ± 3.4			0.0152	29	0.51
*p*_SW_ (SW)		0.3197			0.3687					
ΔBS(cm^2^)		−0.10 ± 0.05			−0.11 ± 0.07			0.6058	1926	0.01
*p*_SW_ (SW)		0.2949			0.8733					
ΔBS%		−7.7 ± 3.5			−9.7 ± 5.8			0.4234	301	0.04
*p*_SW_ (SW)		0.8929			0.7720					
**secondary predictor: molars**										
	**N = 12**		***p*_Wp_** **times**	**N = 19**		***p*_Wp_** **times**	***p*_Wu_** ***ltr* vs. *con***		***ltr* vs. *con***	
**Time X**	**T0**	**T1**	**T0 vs. T1**	**T0**	**T1**	**T0 vs. T1**	**T0**	**T1**	**sample size**	**power**
V (cm^3^)	1.37 ± 0.30	1.07 ± 0.28	**0.0005**	1.35 ± 0.21	0.75 ± 0.16	**0.0001**	0.7000	**0.0015**		
*p*_SW_ (SW)	0.5803	0.0831		0.6063	0.7979					
BS(cm^2^)	1.56 ± 0.43	1.47 ± 0.45	**0.0039**	1.80 ± 0.39	1.71 ± 0.42	**0.0003**	0.1618	0.2647		
*p*_SW_ (SW)	0.5858	0.4003		0.6182	0.6248					
ΔV (cm^3^)		−0.30 ± 0.10			−0.59 ± 0.10			**<0.0001**	6	1.00
*p*_SW_ (SW)		0.3988			0.3843					
ΔV%		−22.3 ± 8.4			−44.3 ± 5.8			**<0.0001**	5	1.00
*p*_SW_ (SW)		0.0282			0.6582					
ΔBS(cm^2^)		−0.09 ± 0.08			−0.10 ± 0.07			0.8233	2726	0.01
*p*_SW_ (SW)		0.2942			0.3087					
ΔBS%		−6.2 ± 5.2			−6.0 ± 4.9			0.9838	31268	0.01
*p*_SW_ (SW)		0.0934			0.1476					
***p*_Wu_ between bicuspids and molars**										
**Time X**	**T0**	**T1**		**T0**	**T1**					
V (cm^3^)	**0.0096**	**0.0048**		**0.0004**	**0.0004**					
BS(cm^2^)	0.1425	0.2316		**0.0004**	**0.0004**					
ΔV (cm^3^)		**0.0010**			**0.0025**					
ΔV%		**0.0008**			**0.0063**					
ΔBS(cm^2^)		0.6712			0.4029					
ΔBS%		0.5118			0.1045					

## Data Availability

Additional data may be available if requested to the institute.

## Abbreviations

LTETs	Less Traumatic Extraction Techniques
.stl	stereolithographic
VOI	Volume Of Interest
V	Volume of alveolar ridge
BS	Basal Surface
*ltr*	less traumatic tooth extraction group
*con*	conventional tooth extraction group

## Appendix A

[x, y, z, c] = stlread(‘FILENAME OF THE STL’);

% voxel dimension (Paolo Toti April 2019)

Dvox = 0.3

Sz = size(c); meshXYZ = zeros(Sz(2),3,3);

xx = reshape(x,3*Sz(2),1); Xmin = min(xx); Xmax = max(xx);

yy = reshape(y,3*Sz(2),1); Ymin = min(yy); Ymax = max(yy);

zz = reshape(z,3*Sz(2),1); Zmin = min(zz); Zmax = max(zz);

Dim = [XminXmaxZminZmaxYminYmax];

x1 = (x − Xmin)/Dvox; y1 = (y − Ymin)/Dvox; z1 = (z − Zmin)/Dvox;

xx1 = reshape(x1,3*Sz(2),1); yy1 = reshape(y1,3*Sz(2),1); zz1 = reshape(z1,3*Sz(2),1);

dimx = round(max(xx1)); dimy = round(max(yy1)); dimz = round(max(zz1));

for i = 1:Sz(2)

meshXYZ(i,:,:) = [x1(:,i)’;y1(:,i)’;z1(:,i)’];

end

[faces,vertices] = CONVERT_meshformat(meshXYZ);

FV1 = struct(‘vertices’,vertices,’faces’,faces);

J1 = polygon2voxel(FV1,[dimx, dimy, dimz],’none’); J2 = imfill(J1,’holes’); patch(x,y,z,c)

patch(isosurface(J2,0.8),’facecolor’,[0 0 1],’edgecolor’,’none’), camlight;view(3)

axis([0dimx0 dimy 0dimz ])

fileCount = 1; sequenceStartNo = 1; sequenceEndNo = dimy; finalZsectional = round(dimy/2);

path2 = ‘FINAL PATH FOR DCM’

path3 = ‘METAFILE PATH FOR DCM’

basename = ‘IM’; fileExtension = ‘.dcm’; Nmin = sequenceStartNo; Nmax = sequenceEndNo;

D001 = zeros(280,280,sequenceEndNo); DFT = size(J2)

for i = 1:dimz

dh = DFT(1); dj = DFT(2);

for h = 1:dh;

for j = 1:dj;

D001(h,j,i) = J2(h,j,i);

end

end

end

imagesc(D001(:,:,int8(dimz./2)),[0 1]); colormap(gray);

for i = sequenceStartNo:sequenceEndNo

if i< 10

sequenceNo = strcat(‘000′,num2str(i));

elseif ((10 <= i ) & (i< 100))

sequenceNo = strcat(‘00′,num2str(i));

elseif ((100 <= i ) & (i<1000))

sequenceNo = strcat(‘0′,num2str(i));

elseif 1000 <i

error(‘More than 1000 files selected’)

end

filename2 = strcat(path2,basename,sequenceNo,’.dcm’);

filename3 = strcat(path3,basename,sequenceNo,fileExtension);

metadata = dicominfo(filename3);

D002 = D001.*2000; D003(:,:,i) = imrotate(D002(:,:,i),0,’bilinear’,’crop’); X017(:,:,i) = int16(D003(:,:,i) − 1);

dicomwrite(X017(:,:,i),filename2, metadata)

if fileCount == 1

dicomHeaderInfo = dicominfo(filename3)

isotropicVoxelDimension = dicomHeaderInfo.PixelSpacing(1);

end

fileCount = fileCount + 1;

end

## Appendix B

% ssA, ssB, ssC … are data matrices at the different time points

threshold = 1000

ppA = ones(280,280,sequenceEndNo);

for i = 1:sequenceEndNo

d = 280; for h = 1:d; for j = 1:d;

if ssA(h,j,i) > threshold

ppA(h,j,i) = ssA(h,j,i);

end

end

end

end
